# Establishment of a model for predicting delayed post-polypectomy bleeding: A real-world retrospective study

**DOI:** 10.3389/fmed.2022.1035646

**Published:** 2022-10-19

**Authors:** Yu Lu, Xiaoying Zhou, Han Chen, Chao Ding, Xinmin Si

**Affiliations:** Department of Gastroenterology, The First Affiliated Hospital of Nanjing Medical University, Nanjing, China

**Keywords:** colorectal neoplasms, endoscopy, gastrointestinal hemorrhage, adverse event, nomogram

## Abstract

**Background:**

Delayed post-polypectomy bleeding (DPPB) is the most common complication which occurs within 30 days after polypectomy, it has become rather common with the widespread of colorectal cancer screening. It is important to clarified predictors of DPPB and identify patients at high risk.

**Materials and methods:**

This was a real-world retrospective study based on medical records from The First Affiliated Hospital of Nanjing Medical University. Cases of patients who underwent colonoscopic polypectomy between January 2016 and December 2020 were reviewed to identify risk factors of DPPB. We use the LASSO-Logistic regression analysis model to identify independent predictors and create a predictive model. The model finally got visualized by developing a nomogram.

**Results:**

Colonoscopic polypectomy was done on 16,925 patients in our study. DPPB occurred in 125 (0.74%) of these instances. In multivariate analysis, age, sex, hypertension, polyp location, polyp size, and operative modality were found to be independent risk factors and were integrated for the construction of a nomogram. The model’s C-index is 0.801 (95%CI: 0.761–0.846). We also found polyps located at the right semicolon and polyp ≥ 1 cm associated with active bleeding under the therapeutic colonoscopy.

**Conclusion:**

Young age, male, hypertension, polyp ≥ 1 cm, proximal colon location and operative modality were finally identified as significant predictors of DPPB. We developed and validated a nomogram which performs well in predicting the incidence of DPPB, the model we established can be used as a valuable screening tool to identify patients who are at high risk of bleeding.

## Introduction

Colorectal polyp is a common intestinal mucosal disease, which can develop into colorectal cancer (CRC) in the adenoma–carcinoma sequence (1). The early resection of precancerous polyps could effectively decrease the incidence and mortality of CRC (2). While colonoscopic polypectomy has become the preferred treatment for less injury and faster recovery, it still carries some serious complications such as bleeding, perforation, and post-polypectomy coagulation syndrome (3). Polypectomy hemorrhage can be divided into immediate polypectomy bleeding (IPB) and delayed post-polypectomy bleeding (DPPB), it is more serious in the latter case as patients cannot receive endoscopic hemostasis promptly (4). Some DPPB might gradually be controlled with the usage of hemostatic, while in most cases, patients with DPPB require blood transfusion or therapy under a repeat colonoscopy, sometimes, even surgical treatment.

To reduce the occurrence of bleeding events, preventive measures should be taken based on the risk assessment of DPPB. Although numerous factors were demonstrated relating to DPPB, it is still difficult for clinicians to make comprehensive assessments. In previous studies, some scoring systems had been constructed to evaluated patients at high risk of DPPB (5, 6). Nomogram is a commonly used tool in clinical research which shows better performance in quantifying variables (7). Individualized prognosis assessment can be realized in diverse situations by utilizing algorithms to integrate several variables and directly displaying the results in form of graphics. This study sought to investigate the predictive factors of DPPB, and firstly develop a risk nomogram to distinguish patients with high-risk of DPPB.

## Materials and methods

### Study design and data source

**FIGURE 1:**
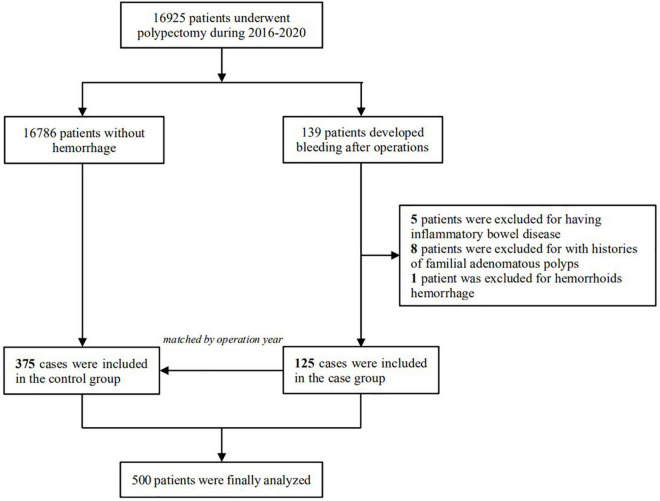
Selection of the study population, 125 patients who developed delayed post-polypectomy bleeding and 375 patients randomly selected from the same cohort were finally included in the study.

This study retrospectively examined consecutive patients who underwent colonoscopic polypectomy at the First Affiliated Hospital of Nanjing Medical University from January 2016 to December 2020. Patients with DPPB were defined as those who experienced lower gastrointestinal bleeding within 30 days after the procedure, they all received additional endoscopies for investigation and hemostasis. The control group was chosen at random from patients without any complications, matched at a 1:3 ratio with the cases according to the year of endoscopy (Figure 1). The study had been approved by the Institutional Review Board of the First Affiliated Hospital of Nanjing Medical University (NO. 2021SR376). Owing to the retrospective design, the ethics committees waived the need for individual informed consent for study participation.

Inclusion criteria: (1) have been diagnosed with colorectal polyps by endoscopic and pathological examination; (2) developed DPPB as defined; (3) with normal coagulation function. Exclusion criteria: (1) combined with hematologic diseases; (2) have a history of inflammatory bowel disease or familial adenomatous polyps; (3) with severe combined heart, lung, or brain diseases or tumors elsewhere; (4) gastrointestinal hemorrhage caused by other disease; (5) without complete clinical data or lack of pathological information.

### Treatment method

Polyethylene glycol solution was used to clean the intestinal canal before the endoscopy. Patients who use antiplatelets or anticoagulants medication were instructed to have a 5–7 days cessation before the procedure and restart the prescription 5 days after polypectomy. During the operation, operators would use the opening width of the biopsy forceps to estimate the diameter of the polyp, then different resection methods were used depending on the size and shape of the polyp. After that, patients would be observed at the hospital for 1–2 days and given hemostatic drugs like Carbazochrome Sodium Sulfonate (CCSS) and hemocoagulase injection, they were closely observed for hemoglobin level and bleeding-related complications such as melena and hematochezia. Patients were advised to rest for at least 2 weeks and have a liquid diet at discharge. For those who suffered post-polypectomy bleeding, we performed another colonoscopy to look for the bleeding site and give relevant hemostatic therapies.

### Observation index

Possible related factors include age, sex, Body Mass Index (BMI), basic disease of patients, the number, size, location, morphology, and pathologic staging of polyps, as well as physician experience, bowel cleanliness, operative modality, and hemorrhage during the operation. For patients with DPPB, we also observed the status of the lesions and hemoclip attachment during the therapeutic endoscopy. Location of the polyp was classified as the right semicolon (ileocecal junction, ascending colon, and transverse colon) and left semicolon (descending colon, sigmoid colon, and rectum). Polyps were divided into sessile polyps and pedunculated polyps by morphologic diagnosis. Bowel preparation status was estimated based on the Boston Bowel preparation scale (8). Operative modalities included argon plasma coagulation (APC), endoscopic mucosal resection (EMR), and endoscopic submucosal dissection (ESD).

### Statistical analyses

Statistical analyses were performed using R software (R version 4.1.2). The categories variables were expressed in percentages and assessed using χ^2^ test or the Fisher exact test, while continuous variables were expressed as mean ± standard deviation when normally distributed, analyzed using *t*-test. Variables with *P*-value < 0.10 in univariate analyses were further included in the least absolute shrinkage and selection operator (LASSO) regression analysis by using the “glmnet” package. Subsequently, multivariable logistic regression analysis was applied to build a predictive model based on screened risk factors above. The results were expressed with odds ratios (ORs) and 95% confidence interval (CI), differences were considered statistically significant when *P* < 0.05.

Additionally, we constructed a nomogram to visualize the model using the “rms” package. The bootstrap repeated sampling method was used to conduct the internal verification. Decision curve analysis (DCA) was performed to verify the clinical practicality of the model. Besides, by calculating the C-index and charting a ROC curve, we examine the performance and discriminative ability of the predictive model.

## Results

A total of 16,925 patients received endoscopic colorectal polypectomy in our center from January 2016 to December 2020. We identified 139 patients who developed postoperative bleeding, and 125 of them who met the inclusion/exclusion criteria were enrolled in the case group. Patients with postoperative hemorrhage received a repeat colonoscopy to search for the bleeding spot and give corresponding treatment, only 9 people bled again even after hemostasis, the rest were successfully treated under endoscopy.

### Baseline information of included patients

The control group, selected from patients without delayed hemorrhage, was matched with the bleeding group according to operation year in a ratio of 3:1. Baseline characteristics of cases and controls revealed that patients with DPPB were younger and have a higher proportion of males as compared to the controls. A significant increase of blood pressure level was found in case group (*P* = 0.001), while the incidence of DPPB shows little relationship with the presence of other chronic diseases which includes diabetes, coronary heart disease, chronic liver or kidney disease (*P* > 0.05). No significant difference was seen between cases and controls in terms of coagulation function (Table 1).

**TABLE 1:** Baseline characteristics of study patients.

	Control group (*n* = 375)	Case group (*n* = 125)	*P*-value
Age, (years)	58.76 ± 11.11	55.73 ± 11.80	0.010
Gender (male), *n* (%)	241 (64.3)	104 (83.2)	<0.001
Body Mass Index, (Kg/m^2^)	24.19 ± 3.17	24.09 ± 3.26	0.768
**Complications, *n* (%)**			
Hypertension			0.001
Normal	251 (67.0)	61 (48.8)	
Prehypertension	104 (27.7)	51 (40.8)	
Hypertension stage 1	20 (5.3)	13 (10.4)	
Diabetes	30 (8.0)	16 (12.8)	0.108
Coronary disease	12 (3.2)	6 (4.8)	0.307
Chronic liver disease	10 (2.7)	2 (1.6)	0.736
Chronic renal disease	5 (1.3)	2 (1.6)	1
Use of antiplatelets/anticoagulants, *n* (%)	23 (6.1)	10 (8.0)	0.467
**Coagulation function**			
PT, (s)	11.75 ± 0.82	11.76 ± 0.72	0.815
INR	1.02 ± 0.06	1.02 ± 0.07	0.795
APTT, (s)	29.00 ± 2.49	29.14 ± 3.07	0.625
TT, (s)	18.16 ± 1.03	18.17 ± 0.95	0.909

PT, prothrombin time; INR, international normalized ratio; APTT, activated partial thromboplastin time; TT, thrombin time.

Parameters shown as mean ± standard deviation were analyzed using independent *t*-test. The other were analyzed using Chi-Square test.

### Polyp-related factors

The characteristics of polyps are displayed in Table 2. DPPB occurred in 78 of the 362 polyps (21.54%) in the left semicolon and 47 of the 138 polyps (34.05%) in the right semicolon, with a statistically significant difference (*P* = 0.004). In regards to polyp morphology, the bleeding rate of pedunculated polyps was much higher than sessile polyps (*P* < 0.001). 1–2 cm polyps and ≥ 2 cm polyps have a higher proportion in the case group when compared with the controls (42.4: 17.9%; 17.6: 5.3%, *P* < 0.001).

**TABLE 2:** Comparison of polyp-related factors of delayed post-polypectomy bleeding in two groups.

	Control group (*n* = 375)	Case group (*n* = 125)	*P*-value
Number of polyps, *n* (%)			0.221
<3	142 (37.9)	41 (32.8)	
3–5	89 (23.7)	25 (20.0)	
≥5	144 (38.4)	59 (47.2)	
Polyp size, *n* (%)			<0.001
<1 cm	288 (76.8)	50 (40.0)	
1–2 cm	67 (17.9)	53 (42.4)	
≥ 2 cm	20 (5.3)	22 (17.6)	
Polyp location, *n* (%)			0.004
Left semicolon	284 (75.7)	78 (62.4)	
Right semicolon	91 (24.3)	47 (37.6)	
Polyp morphology, *n* (%)			<0.001
Sessile polyp	271 (72.3)	56 (44.8)	
Pedunculated polyp	104 (27.7)	69 (55.2)	
Pathology, *n* (%)			<0.001
Hyperplastic polyp	110 (29.4)	14 (11.2)	
Adenoma	239 (63.7)	90 (72.0)	
Adenocarcinoma	26 (6.9)	21 (16.8)	

Parameters were analyzed using Chi-Square test.

### Operation-related factors

The scores of bowel preparation showed no significant difference between the two groups, so did operators’ experience (Table 3). Different resection methods had been taken according to the size and morphology of the polyps, and we found along with the complexity of polypectomy increased, the incidence of DPPB also increase. Sometimes, intraoperative hemorrhage happened due to the damage of small submucosal vessels when removed the polyps, 12.0% of polyps in the bleeding group have this phenomenon, while in the control group, only 4.5% of polyps bled during polypectomy (*P* = 0.003).

**TABLE 3:** Comparison of operation-related factors of delayed post-polypectomy bleeding in two groups.

	Control group (*n* = 375)	Case group (*n* = 125)	*P*-value
Boston Bowel preparation Scale	7.54 ± 1.01	7.37 ± 1.15	0.107
Operator experience, *n* (%)			0.479
5–10 years	55 (14.7)	21 (16.8)	
10–15 years	90 (24.0)	35 (28.0)	
>15 years	230 (61.3)	69 (55.2)	
Operative modality, *n* (%)			<0.001
APC	104 (27.7)	7 (5.6)	
EMR	263 (70.1)	106 (84.8)	
ESD	8 (2.2)	12 (9.6)	
Intraoperative bleeding, *n* (%)			0.003
No	358 (95.5)	110 (88.0)	
Yes	17 (4.5)	15 (12.0)	

APC, argon plasma coagulation; EMR, endoscopic mucosal resection; ESD, endoscopic submucosal dissection.

Parameters shown as mean ± standard deviation were analyzed using independent *t*-test. The other were analyzed using Chi-Square test.

### Identification of factors with predictive value

**FIGURE 2:**
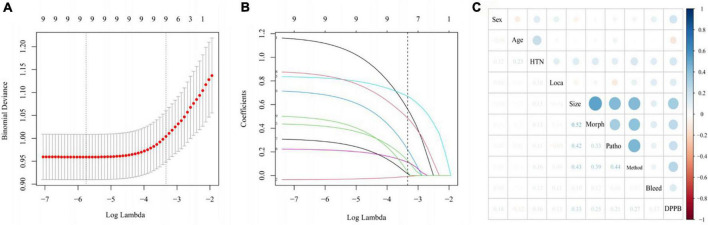
Variable selection using LASSO regression model. **(A)** Tuning parameter (λ) selection in the LASSO model used 10-fold cross-validation via minimum criteria. The lower horizontal coordinate shows the logarithmic value of λ, while the upper horizontal coordinate represents the number of variables with non-zero regression coefficient entering the model. **(B)** LASSO coefficient profiles of the 9 potential predictors. **(C)** Correlation between all variables in the LASSO model visualized by the diameters of the spots.

Nine variables were significantly associated with DPPB, according to the results of the univariate analysis. We applied a LASSO regression analysis based on these variables, as shown in Figure 2. The optimal λ value had been selected by 10 times cross-validation, and dotted vertical lines were drawn based on the minimum criteria and one standard error of the criteria (Figure 2A). All these nine variables were with non-zero coefficients in the LASSO regression analysis (Figure 2B).

### Establishment and assessment of predictive model

Six of the nine variables mentioned above showed significant statistical differences in multivariable logistic regression analysis, they were then selected for the final model (Table 4). These predictors included age (*P* = 0.003, OR = 0.968, 95%CI: 0.948–0.989), sex (*P* < 0.001, OR = 3.154, 95%CI: 1.734–5.737), hypertension (*P* = 0.015, OR = 1.903, 95%CI: 1.130–3.203), polyp location (*P* = 0.003, OR = 2.116, 95%CI: 1.289–3.474), polyp size (*P* < 0.001, OR = 4.035, 95%CI: 2.369–6.872), and operative modality (*P* = 0.029, OR = 2.613, 95%CI: 1.103–6.187).

**TABLE 4:** Multiple logistic regression analysis for risk factors of delayed post-polypectomy bleeding.

	OR	95%CI	*P*-value
**Sex**			
Female	1	–	–
Male	3.154	1.734–5.737	<0.001
Age	0.968	0.948–0.989	0.003
**Hypertension**			
Normal	1	–	–
Prehypertension	1.903	1.130–3.203	0.015
Hypertension stage 1	2.051	0.860–4.888	0.105
**Polyp location**			
Left semicolon	1	–	–
Right semicolon	2.116	1.289–3.474	0.003
**Polyp size**			
<1 cm	1	–	–
1–2 cm	4.035	2.369–6.872	<0.001
≥2 cm	5.634	2.206–14.387	<0.001
**Operative modality**			
APC	1	–	–
EMR	2.613	1.103–6.187	0.029
ESD	4.958	1.074–22.883	0.040

OR, odds ratio; CI, confidence interval.

**FIGURE 3:**
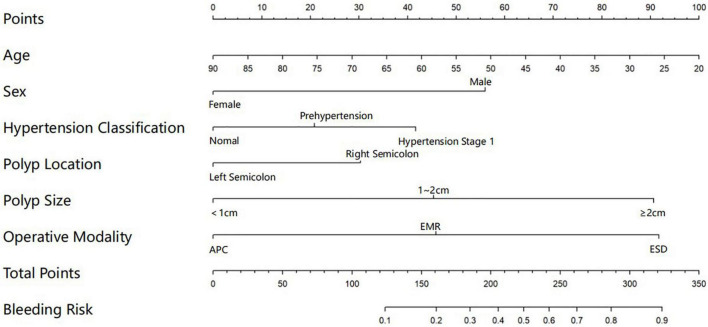
Nomogram for predicting delayed post-polypectomy bleeding (DPPB) in patients who receive colonoscopic polypectomy. Each variable has matched points assigned to a given magnitude of the variable, after calculating the total score, a risk score of DPPB can be found.

**FIGURE 4:**
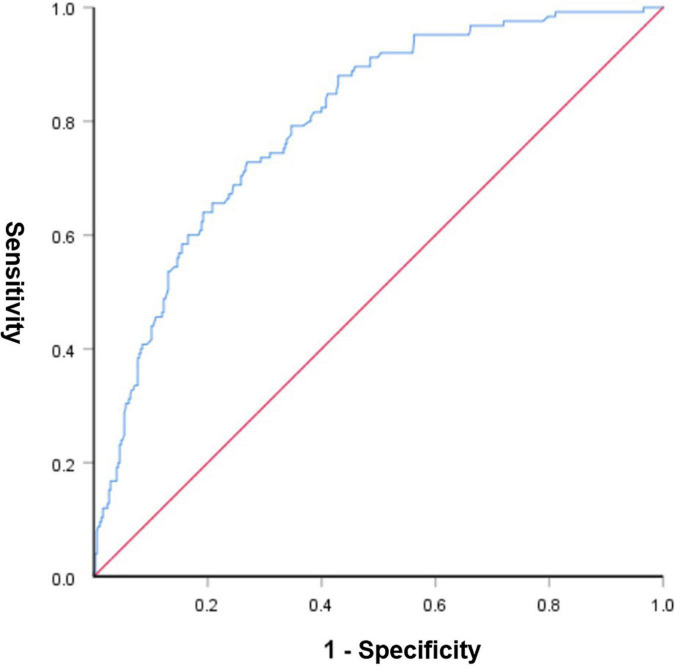
Receiver operating characteristic curve of the predictive nomogram. The area under the ROC was 0.801, demonstrating good discriminative ability of the model.

**FIGURE 5:**
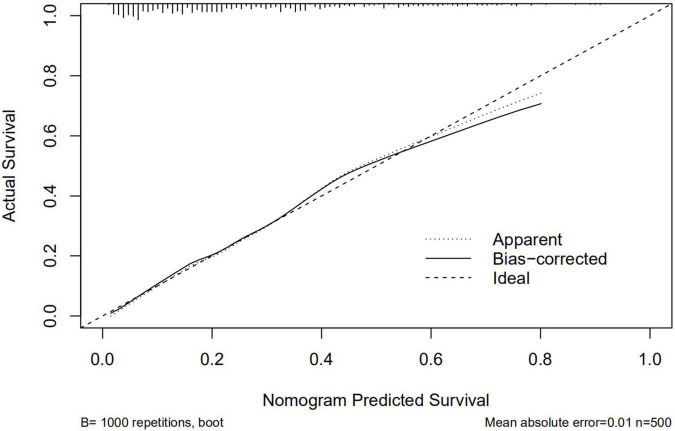
Calibration curves of the predictive nomogram. The x-axis and the y-axis represent the predicted probability and the actual probability of delayed post-polypectomy bleeding, respectively.

**FIGURE 6:**
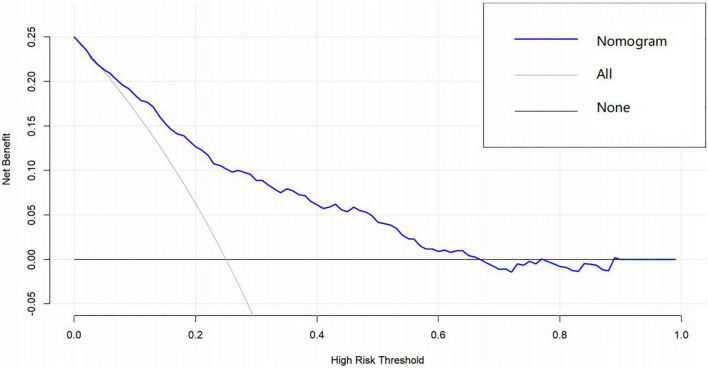
Decision curve analysis of the predictive nomogram. The x-axis and the y-axis represent the net benefit and high-risk threshold, respectively.

A graphic nomogram containing all independent risk factors had been constructed (Figure 3). The c-index of the model was 0.801 (95%CI: 0.761–0.846), showing nice discrimination (Figure 4). The calibration curves (Figure 5) demonstrated adequate agreement between predicted probability and actual observation. What is more, result of decision curve analysis (Figure 6) displayed adequate net clinical benefit, suggesting that the model had good application value.

### Occurring time of delayed post-polypectomy bleeding

**FIGURE 7:**
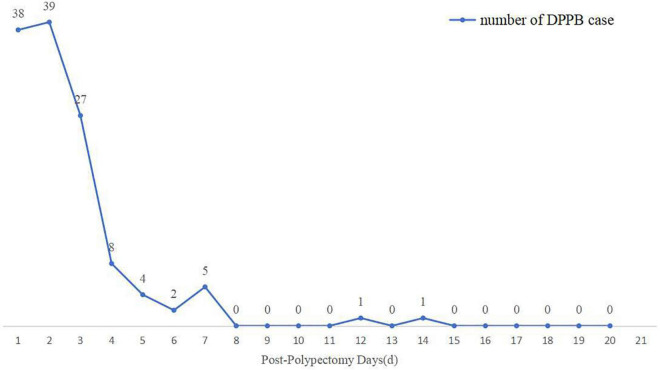
The number of cases of delayed post-polypectomy bleeding (DPPB) and the time interval between completion of polypectomy and occurrence of DPPB.

The mean time interval between polypectomy and occurrence of DPPB was 2.35 days. Bleeding happened within 24 h after polypectomy in 38 patients, 24–48 h in 39 patients, 48 h–7 days in 46 patients, only 2 patients bleeding over a week, as shown in Figure 7. Four variables were finally included in multivariate analysis, Nevertheless, none of these factors were identified as independent predictors (Table 5).

**TABLE 5:** Univariable analysis and multivariable logistic analysis for factors associated with delayed post-polypectomy bleeding after 48 h.

Univariate	DPPB within 48 h (*n* = 77)	DPPB after 48 h (*n* = 48)	*P*-value
Age, years	56.17 ± 11.38	55.02 ± 12.54	0.599
Male sex, *n* (%)	67 (87.0)	37 (77.1)	0.149
Body Mass Index, (Kg/m^2^)	24.47 ± 3.41	23.49 ± 2.95	0.103
**Complications, *n* (%)**			
Hypertension			0.377
Normal	34 (44.2)	27 (56.3)	
Prehypertension	35 (45.5)	16 (33.3)	
Hypertension stage 1	8 (10.3)	5 (10.4)	
Diabetes	10 (13.0)	6 (12.5)	0.937
Coronary disease	5 (6.5)	1 (2.1)	0.489
Chronic liver disease	2 (2.6)	0 (0)	0.523
Chronic renal disease	1 (1.3)	1 (2.1)	1.000
Use of antiplatelets/anticoagulants, *n* (%)	5 (6.5)	5 (10.4)	0.655
Polyp size, *n* (%)			0.082
<1 cm	34 (44.2)	16 (33.3)	
1–2 cm	34 (44.2)	19 (39.6)	
≥2 cm	9 (11.6)	13 (27.1)	
Polyp location, *n* (%)			0.022
Left semicolon	42 (54.5)	36 (75.0)	
Right semicolon	35 (45.5)	12 (25.0)	
Polyp morphology, *n* (%)			0.354
Sessile polyp	37 (48.1)	19 (39.6)	
Pedunculated polyp	40 (51.9)	29 (60.4)	
Pathology, *n* (%)			0.014
Hyperplastic polyp	9 (11.7)	5 (10.4)	
Adenoma	61 (79.2)	29 (60.4)	
Adenocarcinoma	7 (9.1)	14 (29.2)	
Operative modality, *n* (%)			0.098
APC	4 (5.2)	3 (6.3)	
EMR	69 (89.6)	37 (77.0)	
ESD	4 (5.2)	8 (16.7)	
Boston Bowel preparation Scale	7.51 ± 1.06	7.17 ± 1.28	0.110
Operator experience, *n* (%)			0.229
0–5 years	12 (15.6)	9 (18.8)	
5–10 years	18 (23.4)	17 (35.4)	
>10 years	47 (61.0)	22 (45.8)	
Hemoclip usage, *n* (%)			0.858
Yes	71 (92.2)	43 (89.6)	
No	6 (7.8)	5 (10.4)	
Hemoclip missing, *n* (%)			0.145
No	59 (76.6)	31 (64.6)	
Yes	18 (23.4)	17 (35.4)	
Intraoperative bleeding, *n* (%)			0.205
No	70 (90.9)	40 (83.3)	
Yes	7 (9.1)	8 (16.7)	

**Multivariate**	**OR**	**95%CI**	***P*-value**

**Polyp size**			
<1 cm	1	–	–
1–2 cm	1.046	0.418–2.617	0.923
≥2 cm	1.383	0.326–5.867	0.660
**Polyp location**			
Left semicolon	1	–	–
Right semicolon	0.499	0.212–1.172	0.111
**Pathology**			
Hyperplastic polyp	1	–	–
Adenoma	0.826	0.240–2.840	0.761
Adenocarcinoma	2.167	0.445–10.561	0.339
**Operative modality**			
APC	1	–	–
EMR	0.624	0.118–3.315	0.580
ESD	1.304	0.126–14.384	0.829

DPPB, delayed post-polypectomy bleeding; APC, argon plasma coagulation; EMR, endoscopic mucosal resection; ESD, endoscopic submucosal dissection; OR, odds ratio; CI, confidence interval.

Parameters shown as mean ± standard deviation were analyzed using independent *t*-test. Operative modality was analyzed using Fisher’s exact test. The other were analyzed using Chi-Square test. Parameters with *P*-value < 0.10 in univariate analyses were included in multivariable logistic analysis.

### Status of polypectomy sites after delayed post-polypectomy bleeding

**FIGURE 8:**
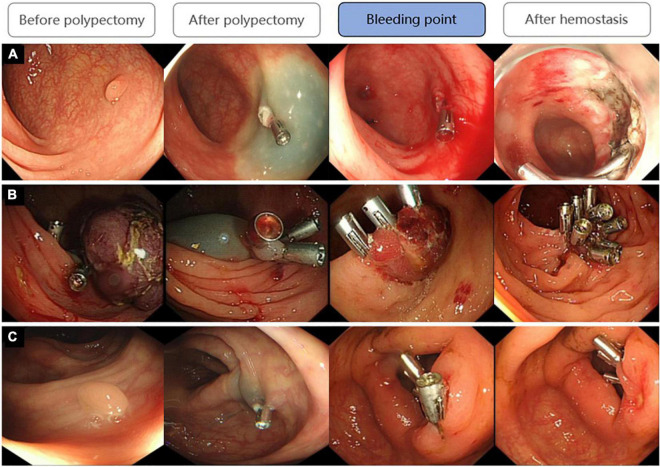
Three states of bleeding points after DPPB: **(A)** Active bleeding; **(B)** Had formed blood clots or scabs covering the wound; **(C)** No obvious bleeding spot but some old bloodstain had been observed.

For patients with DPPB, we observed a total of 68 patients had active bleeding under the endoscopy, 41 patients had formed blood clots or scabs on the surface of lesions which maybe the reason for well-controlled hemorrhage, clean-based ulcer was found at the polypectomy site in 9 patients, none obvious bleeding spot was discovered in the remaining 7 patients (Figure 8). Active bleeding was associated with size, morphology and location of polyps. Hemoclips were found to have fallen off prematurely at some polypectomy sites. Although this phenomenon happened more frequently on patients had active bleeding under the endoscopy (32.4:22.8%), the difference is not statistically significant (*P* = 0.236). In multivariable logistic regression analysis, polyp size and polyp location had been discovered as independent predictors of patients with active bleeding (Table 6).

**TABLE 6:** Univariable analysis and multivariable logistic analysis of factors associated with active bleeding during the second colonoscopy.

Univariate	No active bleeding (*n* = 57)	Active bleeding (*n* = 68)	*P*-value
Age, years	55.74 ± 11.04	55.72 ± 12.49	0.994
Male sex, *n* (%)	49 (86.0)	55 (80.9)	0.449
Body Mass Index, (Kg/m^2^)	23.75 ± 3.47	23.37 ± 3.07	0.298
**Complications, *n* (%)**			
Hypertension			0.290
Normal	26 (45.6)	35 (51.5)	
Prehypertension	27 (47.4)	24 (35.3)	
Hypertension stage 1	4 (7.0)	9 (13.2)	
Diabetes	7 (12.3)	9 (13.2)	0.874
Coronary disease	4 (7.0)	2 (2.9)	0.521
Chronic liver disease	1 (1.8)	1 (1.5)	1.000
Chronic renal disease	0 (0)	2 (2.9)	0.500
Use of antiplatelets/anticoagulants, *n* (%)	5 (8.8)	5 (7.4)	1.000
Hemoglobin decrease > 2 g/dL, *n* (%)	13 (22.8)	20 (29.4)	0.404
Polyp size, *n* (%)			0.011
<1 cm	31 (54.4)	19 (27.9)	
1–2 cm	18 (31.6)	35 (51.5)	
≥2 cm	8 (14.0)	14 (20.6)	
Polyp location, *n* (%)			0.017
Left semicolon	42 (73.7)	36 (52.9)	
Right semicolon	15 (26.3)	32 (47.1)	
Polyp morphology, *n* (%)			0.020
Sessile polyp	32 (56.1)	24 (35.3)	
Pedunculated polyp	25 (43.9)	44 (64.7)	
Pathology, *n* (%)			0.329
Hyperplastic polyp	9 (15.8)	5 (7.4)	
Adenoma	39 (68.4)	51 (75.0)	
Adenocarcinoma	9 (15.8)	12 (17.6)	
Operative modality, *n* (%)			0.606
APC	2 (3.5)	5 (7.4)	
EMR	50 (87.7)	56 (82.4)	
ESD	5 (8.8)	7 (10.2)	
Boston Bowel preparation Scale, *n* (%)	7.39 ± 1.10	7.37 ± 1.21	0.929
Operator experience, *n* (%)			0.276
0–5 years	10 (17.5)	11 (16.2)	
5–10 years	12 (21.1)	23 (33.8)	
>10 years	35 (61.4)	34 (50.0)	
Hemoclip usage, *n* (%)			0.992
Yes	52 (91.2)	62 (91.2)	
No	5 (8.8)	6 (8.8)	
Hemoclip missing, *n* (%)			0.236
No	44 (77.2)	46 (67.6)	
Yes	13 (22.8)	22 (32.4)	
Intraoperative bleeding, *n* (%)			0.643
No	51 (89.5)	59 (86.8)	
Yes	6 (10.5)	9 (13.2)	

**Multivariate**	**OR**	**95%CI**	***P* value**

**Polyp size**			
<1 cm	1	–	–
1–2 cm	3.288	1.401–7.715	0.006
≥2 cm	3.009	1.002–9.035	0.050
**Polyp location**			
Left semicolon	1	–	–
Right semicolon	2.645	1.177–5.941	0.018
**Polyp morphology**			
Sessile polyp	1	–	–
Pedunculated polyp	1.613	0.656–3.967	0.298

APC, argon plasma coagulation; EMR, endoscopic mucosal resection; ESD, endoscopic submucosal dissection; OR, odds ratio; CI, confidence interval.

Parameters shown as mean ± standard deviation were analyzed using independent *t*-test. Operative modality was analyzed using Fisher’s exact test. The other were analyzed using Chi-Square test. Parameters with *P*-value < 0.10 in univariate analyses were included in multivariable logistic analysis.

## Discussion

Previous studies have shown that the incidence of DPPB ranged from 0.3 to 1.3% (9–12). The bleeding rate in our center was 0.74%, which was consistent with previous records. Here we discussed relevant risk factors of DPPB from 3 aspects: (1) patient-related factors; (2) polyp-related factors; (3) operation-related factors. By using the Lasso logistic regression algorithm, six independent risk predictors were effectively screened out. These predictors included age, sex, hypertension, polyp location, polyp size and operative modality. The nomogram based on these factors showed good prediction ability and can be used as a quantitative tool to evaluate incidence of DPPB.

In contrast to most of the previous studies, (13, 14) we found young patients are more likely to develop DPPB. This may have relation with their failure to adhere to post-operation advice for dietary and lifestyle adjustment, as most of the young patients have to restart work as soon as possible. The result corresponds with the findings of Niikura et al. (12) and Park et al. (15) who found young patients to be more susceptible to serious DPPB. We also observed poorly controlled hypertension was significantly associated with the increased incidence of DPPB. This finding was consisted with the study by Choung, B. S. et al., which also found a significantly higher proportion of patients with hypertension in the DPPB group than those without bleeding (16). We assume that this may be associated with atherosclerosis and vasoconstriction dysfunction caused by the prolonged excessive pressure of the blood on the vessel wall, which results in poor vasoconstriction at the mucosal lesion of the colon. Furthermore, we also evaluated the effects of anticoagulant and antiplatelet drugs on DPPB. Previous studies have shown that patients who take these drugs are more likely to suffer significant bleeding events (12, 17–19). This study showed no significant difference in antithrombotic users, probably because patients taking these drugs in our center had been asked to have a cessation for more than 5–7 days according to the guideline (20). Although the cessation could effectively reduce the incidence of DPPB, we still should pay attention to possible thromboembolic events at the same time, which requires more relevant researches in future.

Polyp size was identified as the most important risk factor in nearly all previous studies, especially polyps ≥ 10 mm, have several times higher bleeding risk than small polyps (9, 21–23). It has been demonstrated that polyp morphology was also an important risk factor. A 10-year retrospective analysis found pedunculated polyps have a higher bleeding tendency, mostly because the stem of pedunculated polyp usually contains nutrient vessels. These vessels would be injured during the resection, which may increase the risk of immediate and delayed bleeding (24). In addition, polyp location also influences the occurrence of DPPB. The proximal and distal colon have many differences in caliber, histology, blood supply and composition of digestive fluid. Many studies found that polyps located on the right semicolon have a higher bleeding rate compared to those on the left (23, 25, 26). Our study suggests that the incidence of DPPB was 2.116 times higher in polyp located at the proximal semicolon.

Regarding the operation method, we find polyps removed by ESD and EMR had higher risks of bleeding, which complies with the findings of Liu and his colleagues (27, 28). Moreover, we observed patients with intraoperative bleeding seems more susceptible to DPPB. Intraoperative bleeding suggests injury of submucosal vessels during polyp resection, although we can immediately stanch bleeding by argon plasma coagulation or by using hemostatic clips, the possibility of incomplete clamping, early displacement of the clips and rebleeding of the lesions is still existing, leading to an increased risk of DPPB (28).

Next, we conduct subgroup analysis to further investigate patients with DPPB in different conditions. Firstly, we explored possible influence factors associated with hemorrhage over 48 h, when most of the patients had been discharged from the hospital and were difficult to get timely treatment. Although no significant difference was found between patients who appeared bleeding within or over 48 h, they still had some differences in polyp location and polyp pathology. Secondly, for patients with active bleeding under the therapeutic endoscopy, we found polyp ≥ 1 cm and proximal colon location were independent predictors. Therefrom, we suggest patients with these risk factors should receive timely endoscopic therapies to avoid severe adverse events.

Although incidence of DPPB was only 0.74% in this study, given the large and incremental population of patients needing endoscopic polypectomy, it deserves more attention. We built a predictive nomogram model of DPPB for the first time. Compared with the scoring models in former researches, the nomogram could have a better performance in quantifying all the predictors. The model established in this study was concise, it only involves six indicators, which are fully proven related to DPPB in previous researches and easily available in different medical centers. However, this research also contains limitations as a retrospective study in a single center. Besides, we only performed internal verification, external verification is still needed to be conducted in further research. By developing a nomogram to predict DPPB, we provide a simple and visualizable way to calculate the risk of DPPB of each patient. For patients with higher scores, clinicians could take more active measures, such as controlling perioperative blood pressure to a more proper range, prolonging the observation period after operation as well as enhancing hemostasis therapy, in order to reduce the occurrence of clinical bleeding events.

## Data availability statement

The raw data supporting the conclusions of this article will be made available by the authors, without undue reservation.

## Ethics statement

The studies involving human participants were reviewed and approved by the Ethics Committee of Jiangsu Provincial Hospital. Written informed consent for participation was not required for this study in accordance with the national legislation and the institutional requirements.

## Author contributions

All authors listed have made a substantial, direct, and intellectual contribution to the work, and approved it for publication.
